# Genome Sequence and Annotation of *Trichoderma parareesei*, the Ancestor of the Cellulase Producer *Trichoderma reesei*

**DOI:** 10.1128/genomeA.00885-15

**Published:** 2015-08-13

**Authors:** Dongqing Yang, Kyle Pomraning, Alexey Kopchinskiy, Razieh Karimi Aghcheh, Lea Atanasova, Komal Chenthamara, Scott E. Baker, Ruifu Zhang, Qirong Shen, Michael Freitag, Christian P. Kubicek, Irina S. Druzhinina

**Affiliations:** a Research Area Biotechnology and Microbiology, Institute of Chemical Engineering, TU Wien, Vienna, Austria; b Jiangsu Key Lab for Organic Waste Utilization and National Engineering Research Centre for Organic-Based Fertilizers, Nanjing Agricultural University, Nanjing, China; c Environmental Molecular Sciences Laboratory, Pacific Northwest National Laboratory, Richland, Washington, USA; d Department of Biochemistry and Biophysics, Oregon State University, Corvallis, Oregon, USA

## Abstract

The filamentous fungus *Trichoderma parareesei* is the asexually reproducing ancestor of *Trichoderma reesei*, the holomorphic industrial producer of cellulase and hemicellulase. Here, we present the genome sequence of the *T. parareesei* type strain CBS 125925, which contains genes for 9,318 proteins.

## GENOME ANNOUNCEMENT

The filamentous ascomycete *Trichoderma reesei* is used in the biotechnological industry for the production of cellulolytic and hemicellulolytic enzymes and recombinant proteins (1). In nature, *T. reesei* almost exclusively occurs in its sexual form on dead wood (2). Earlier isolations of putative *T. reesei* anamorphs from soil (3, 4) have been shown to be a sympatric sister species that is now named *Trichoderma parareesei* (5). Compared to *T. reesei*, the later taxon has an entirely clonal lifestyle, is considerably more versatile in substrate utilization, and has enhanced mycoparasitic vigor (2, 5).

We sequenced the genome of the type strain of *T. parareesei* CBS 125925 in an Illumina-based whole-genome shotgun sequencing approach delivering 366,865,176 paired reads with an approximate insert size of 350 bp. The acquired sequence reads were assembled into 1,123 contigs using Velvet v1.0.12 (6) with a k-mer length of 75 nucleotides (nt). The resulting genome sequence has an estimated size of 32.0 Mb (*N*_50_, 68,608 bp; *N*_Max_, 286,763 bp; median coverage, 250.5) with a G+C content of 53.8%.

The genome assembly was repeat masked by RepeatMasker (http://www.repeatmasker.org), and the protein-coding genes were predicted by combining ab initio and homology-based approaches. The training set combined self-training GeneMark-ES v2.3f predictions and homology protein alignment using Exonerate. This set was then used to train AUGUSTUS v.27 and SNAP and finally combined by EVidenceModeler (EVM) to yield 9,318 consensus gene models (7), 8,651 (93%) of which had orthologs in *T. reesei*.

### Nucleotide sequence accession numbers.

This whole-genome shotgun project has been deposited at DDBJ/EMBL/GenBank under the accession number LFMI00000000. The version described in this paper is version LFMI01000000.
